# Case Report: Necrotizing granulomas in the central nervous system: sarcoidosis masquerading as neurotuberculosis

**DOI:** 10.3389/fimmu.2025.1653164

**Published:** 2025-10-30

**Authors:** Clothilde Gros, Karolina Hankiewicz, Adrien Carle, Dominique Cazals-Hatem, Mickael Bonnan

**Affiliations:** ^1^ Hôpital Delafontaine, Department of Neurology, Saint-Denis, France; ^2^ Hôpital Beaujon, Department of Neurosurgery, Assistance publique des Hôpitaux de Paris, Université de Paris, Clichy, France; ^3^ Hôpital Beaujon, Department of Pathology, Assistance publique des Hôpitaux de Paris, Université de Paris, Clichy, France

**Keywords:** sarcoidosis, tuberculosis, pathology, granuloma, necrosis

## Abstract

Neurosarcoidosis (NS) can affect patients with or without any systemic involvement. Diagnosis of NS without lung involvement requires (i) a biopsy showing typical non-necrotizing granulomas and (ii) exclusion of tuberculosis by negative culture for M. tuberculosis and PCR amplification techniques. In the absence of microbiological infection, the diagnosis is challenging when granulomas are necrotizing mimicking tuberculosis on histology. We report two cases presenting neurological symptoms and radiological lesions that were concordant with the diagnosis of NS. Nevertheless, brain biopsy showed necrotizing granulomas. Antineutrophil cytoplasmic antibodies (ANCA) showed a negative finding. Given the biopsy results, we started anti-tuberculosis treatment despite negative mycobacterial test results. Lack of improvement suggested the diagnosis of necrotizing sarcoid granulomatosis (NSG). NSG usually presents granulomas, necrosis, and vasculitis, mostly in the lungs. However, these cases presented granulomas with extensive necrosis and vasculitis compatible with NSG strictly limited to the central nervous system. The final diagnosis was NS, as NSG is sometimes understood as presenting a pattern of sarcoidosis, a hypothesis supported by the sustained remission obtained under immunosuppressive treatment. Brain NSG should not rule out the diagnosis of NS and lead to the diagnosis of tuberculosis. NS or NSG should still be evoked, especially if microbiological and immunological investigations are negative and even if the central nervous system is the unique organ involved.

## Introduction

Sarcoidosis is a multisystem granulomatous disease of unknown origin that preferentially affects the lungs but can involve any organ. It affects the central nervous system (CNS) in 5% to 26% of patients with or without any systemic manifestations, and neurological symptoms are isolated in 10% to 28% of cases (1). The diagnosis of neurosarcoidosis (NS) is based on a range of clinical (i.e., meningitis, focal signs, cranial neuropathies, myelopathy), biological (i.e., hypercalcemia, hypergammaglobulinemia, increased angiotensin-converting enzyme [ACE]), and radiological signs with proven histology, while eliminating other differential granulomatous diagnoses such as infections, neoplasia, and vasculitis. Even if a neurological biopsy may be difficult to obtain, the presence of non-necrotizing granulomas remains a diagnostic pillar after ruling out infectious diseases, which can lead to the initiation of steroids or antitumor necrosis factor drugs (2). On the other hand, necrotizing granulomas are highly uncommon in sarcoidosis but provide strong evidence in favor of infection. We report two cases in which necrotizing granulomas on brain biopsy initially obscured the diagnosis.

## Case description

### Case 1

**Figure 1:**
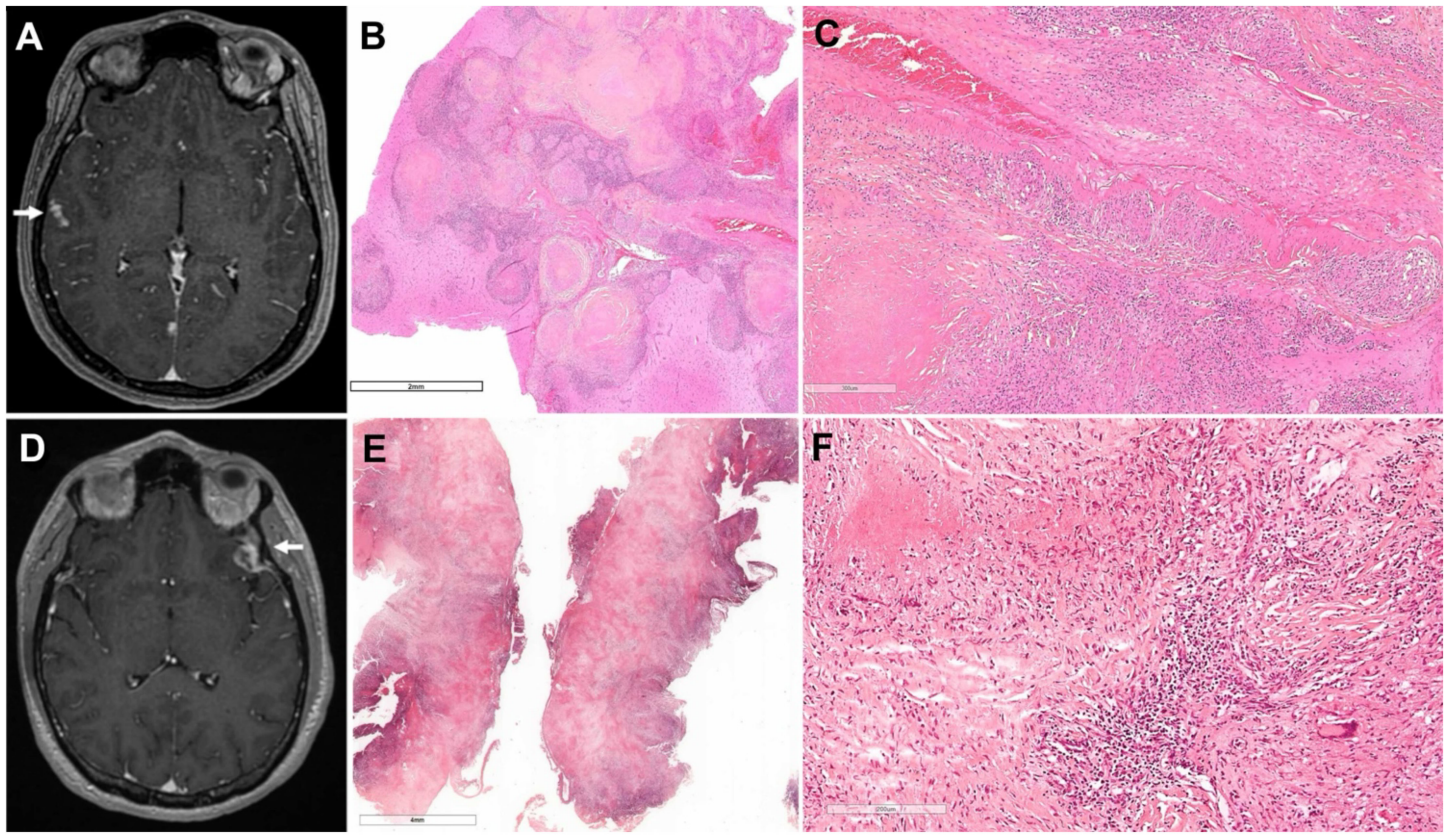
Imaging and histopathological findings at onset. Patient 1 **(A–C)** – **(A)** T1-weighted brain MRI showing multiple leptomeningeal enhancements (right temporal lesion analyzed on histology, arrow). **(B)** Cortical lesion shows an accumulation of necrotic nodules surrounded by fibrosis and lymphocytes. **(C)** Many epithelioid granulomas are observed along an occluded artery at a distance from the nodules. Grocott’s methenamine silver and Ziehl’s colorations were negative (not shown). Patient 2 **(D–F)** – **(D)** T1-weighted brain MRI at onset showing a contrast-enhanced left temporal lesion (left frontal lesion analyzed on histology, arrow). **(E)** Cortical lesion shows diffuse central necrosis surrounded by fibrosis and a rim of chronic lymphoid inflammation. **(F)** Epithelioid and giant cell granulomas form the inflammation around the fibrinoid necrosis. Giemsa, PAS, and Ziehl’s colorations were negative (not shown).

A woman in her 30s without any medical or familial history presented fluctuating left-side numbness and moderate headache. One month later, she was admitted to the emergency unit for acute left central facial paralysis. Brain magnetic resonance imaging (MRI) showed multiple contrast-enhancing T1 hyperintensities of tentorial and infra-tentorial white matter and leptomeningeal contrast-enhancement but no signs of recent ischemic stroke (**Figure 1**; **Supplementary Figure S1**). Her cerebral spinal fluid (CSF) level was 60 cells/µL, mainly lymphocytes with increased proteins (64 mg/dL) and normal glucose. Oligoclonal bands were present and ACE was normal. Interleukin-6 and interleukin-10 levels were not suggestive of lymphoma, and immunophenotyping showed a normal result. Results of bacterial and mycobacterial cultures, *M. tuberculosis* PCR, and test for cryptococcal antigen remained negative. Ocular examination revealed a unilateral posterior uveitis scar. Fluorodeoxyglucose-positron emission tomography revealed intense diffuse hypermetabolism of the tissues of the oral sphere, abnormally pronounced hypermetabolism of the spinal cord, and diffuse unspecific bone marrow hypermetabolism. Spinal MRI demonstrated leptomeningeal contrast enhancement at the level of the terminal cone and lower dorsal medulla. Nasal cavity biopsy revealed one granuloma without necrosis, and a salivary gland biopsy was normal. Blood count, ionogram, liver tests, and calcium had normal results. Other wide-spectrum non-invasive investigations showed normal findings including ACE in blood and CSF, negative antinuclear anti-phospholipids and anti-neuronal antibodies, positive antineutrophil cytoplasmic antibodies (ANCA) without any PR3 or MPO, no hypergammaglobulinemia, negative HCV and HIV serology, protective HBV serology, negative BD-glucans, and normal body CT scan.

Facial palsy and headache abated spontaneously, but control brain MRI showed the persistence of contrast-enhancing intraparenchymal hyperintense FLAIR signals. Right temporal neuromeningeal biopsy revealed a conglomerate of granuloma characterized by epithelioid and Langhans giant cells surrounding eosinophilic acellular necrosis (**Figure 1**), suggesting an active tuberculosis (TB). Ziehl Neelsen’s stain and mycobacterial cultures on CSF and biopsy, QuantiFERON-TB test, and PCR for M. tuberculosis on CSF showed negative findings; chest CT scan also presented normal results. Quadritherapy against TB and steroids were started in January 2022.

**Figure 2:**
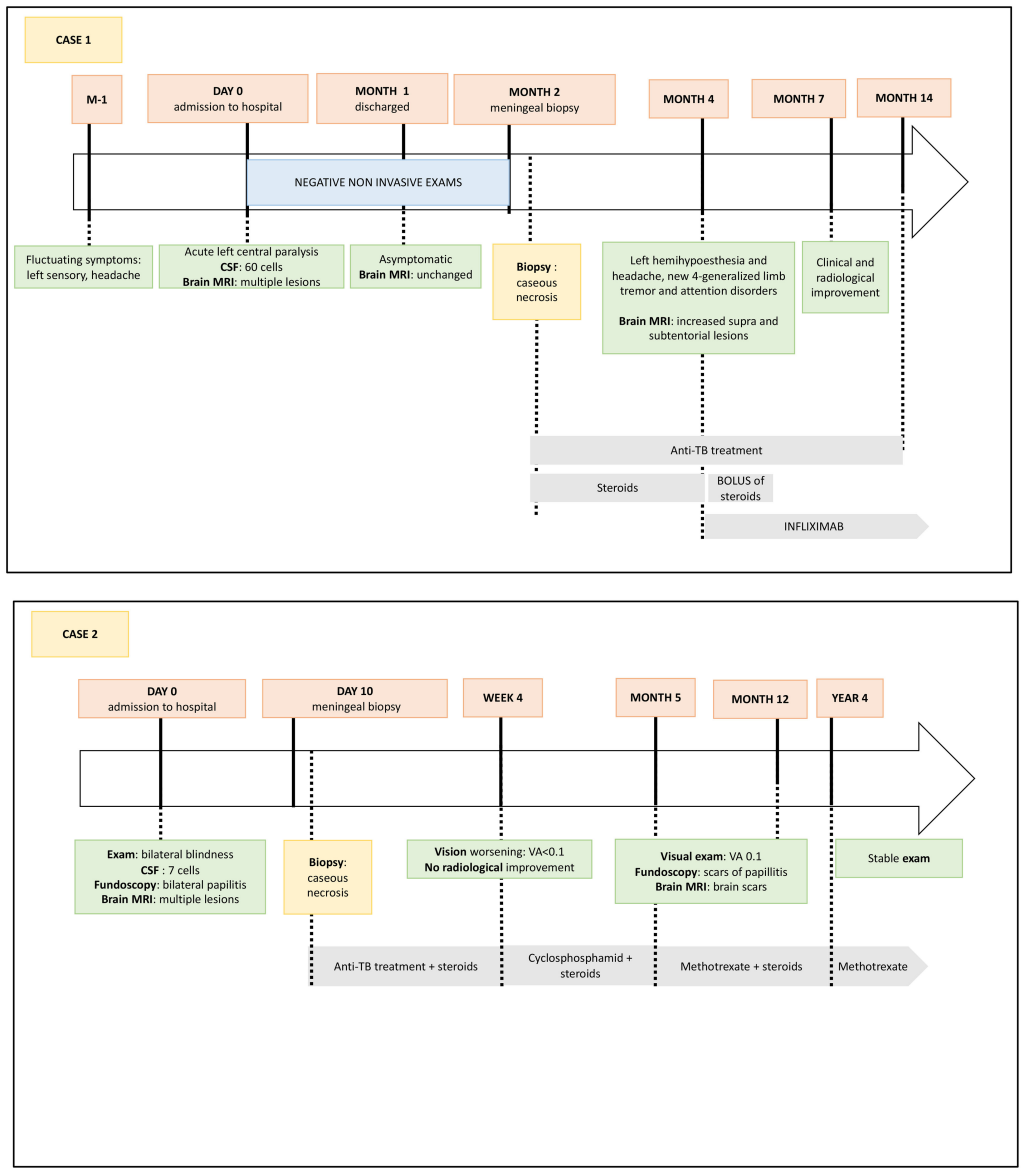
Historical timeline of cases.

After 2 months of treatment, steroids were tapered. Left hemihypoesthesia and headache soon reappeared followed by limb tremor and the development of an attention disorder. Brain MRI showed a larger nodular leptomeningeal contrast enhancement. Findings of further immunological tests remained negative. The patient was diagnosed with immune reconstitution inflammatory syndrome, so the steroid dose was increased again, leading to transient incomplete improvement of headache and general status. Reassessment after 12 months of anti-TB showed the persistence of lymphocytic meningitis and progression of lesions radiologically. The diagnosis was reconsidered, and high-dose steroids and infliximab perfusions (5 mg/kg/month) were administered. Infliximab was chosen given its greater efficacy in severe NS (3). Reassessment during the next 2 years showed major MRI and clinical improvement (**Figure 2**).

### Case 2

A 39-year-old man without any medical or familial history was admitted to the neurology department for neuropapillitis manifested by subacute bilateral blindness. He initially complained of a monolateral decrease in visual acuity that affected the contralateral eye a few weeks later with bilateral blindness. At admission, brain MRI showed left temporal and peri-ventricular neuromeningeal lesions with contrast enhancement (**Figure 1**; **Supplementary Figure S2**). Clinical examination presented normal findings except for the blindness. His CSF level was 7 cells/µL with a discrete increase in protein level (50 mg/dL), normal glucose level, and the presence of oligoclonal bands. Tests for M. tuberculosis PCR, RNA 16S PCR, and cryptococcal antigen all showed negative results.

As non-invasive investigations were not informative (normal blood count, ionogram, liver test, and CRP; negative Bartonella, syphilis, Lyme, and HIV serologies with negative QuantiFERON-TB; negative ANCA and anti-neuronal antibodies; and normal ACE), a left temporal neuromeningeal biopsy was performed. Histology revealed granulomatous inflammation with acellular necrosis suggestive of TB (**Figure 1**) despite negative bacterial and mycobacterial cultures and M. tuberculosis PCR. Despite the initiation of anti-TB treatment and steroids, there was no clinical or radiologic improvement so the diagnosis of sarcoidosis was evoked. After 1 month, the anti-TB treatment was stopped and replaced by high-dose steroids and short-course IV cyclophosphamide (with trimethoprim and sulfamethoxazole as prophylaxis for Pneumocystis jirovecii pneumonia), with a subsequent switch to methotrexate (0.3 mg/kg/week). The size of the brain lesions began to decrease on MRI. After 2 years of methotrexate, the patient’s visual acuity had slightly improved. Fundoscopy showed optic atrophy and no inflammation, and brain MRI only highlighted parenchymal scars, including the frontal one, with no disease activity. Steroids were tapered and discontinued after 4 years of treatment, but methotrexate was continued (**Figure 2**).

## Discussion

These patients illustrate the difficulty in the diagnosis of NS: Both presented with a suggestion of NS, whereas the biopsy led to a diagnosis of tuberculosis due to an unexpected necrotizing granuloma. Given the negative infectious context and immunological tests (especially ANCA), the diagnosis of NS was likely on histology. Additional necrosis confused with infection, mainly tuberculosis and chronic fungal infections (such as histoplasmosis, aspergillosis, and cryptococcosis) and with necrotizing vasculitis such as Wegener’s granulomatosis (4–6). Excluding these latter diagnoses, the lack of improvement with anti-TB treatment was decisive for the diagnosis of NS, characterized by well-circumscribed perivascular epithelioid and giant-cell granulomas generally located at the surface of the brain or meninges. Necrosis is rarely described in sarcoidosis and, when present, is limited, focal, and non-caseous on gross description. Fibrosis replaces the necrosis in chronic cases (2, 7). Necrotizing sarcoid granulomatosis (NSG) has previously been described in the brain, interpreted as a late stage of nodular sarcoidosis in which granulomas distributed along vessels cause fibrosis and secondary ischemic lesions (8). Some authors proposed the term “sarcoidosis with a necrotizing sarcoid granulomatosis pattern” (9).

NSG is very rare and mainly involves the lungs, whereas CNS is usually spared. A review found that in at least 6% of cases of pulmonary NSG, the CNS was also affected, although rarely biopsied (10). Neuromeningeal biopsy was considered too invasive a procedure, so it remains unclear how often necrotizing lesions observed in the lung may also occur in CNS lesions. We reviewed NSG with CNS involvement and found 25 cases including ours (see **Supplementary Table S1**). No systemic sarcoidosis was observed in 21/25. CNS lesions encompassed the range of usual NS lesions without an apparent specific pattern. The biopsy location was in the CNS in 17/24, in the orbit in 2/24, and otherwise in the lung. Pathology showed granuloma in all cases, necrotizing lesions in 21/25 and vasculitis in 6/23. All cases of vasculitis were observed in lung or orbit biopsies.

The present observations of NS are particularly interesting because both patients had no history of known sarcoidosis prior to CNS onset, and both benefited from full histological analysis showing granulomas with necrosis, which was atypical for a sarcoidosis. These patients are still in remission a few years after the diagnosis, and CNS lesions disappeared. As about 30% to 50% of patients with NS relapse, albeit with lower rates under immunosuppressive therapy (11, 12), the diagnosis remains robust even without any relapse. Moreover, the spontaneous remission of an unproven infectious disease, especially TB, can be ruled out.

## Conclusion

Histology of neurosarcoidosis is usually informative when showing non-necrotizing granulomas. The diagnosis is challenging when granulomas are necrotizing in the absence of microbiological infection. Clinicians should be made aware that the diagnosis of NS should not be completely ruled out by the presence of CNS necrotizing lesions.

## Data Availability

The datasets presented in this article are not readily available because of ethical and privacy restrictions. Requests to access the datasets should be directed to the corresponding author.
