# Supplementary material for: Case Report: Necrotizing granulomas in the central nervous system: sarcoidosis masquerading as neurotuberculosis
Source: Front Immunol. 2025 Oct 30;16:1653164. doi: 10.3389/fimmu.2025.1653164 (PMC12611859; doi:10.3389/fimmu.2025.1653164)
Supplement: Supplementary file 2 [file Table1.docx]

**Supplemental file 1 PRISMA flow diagram**

Records identified from PubMed

(n = 176)

Records screened

(n = 176)

Records excluded: not focused on necrotizing lesions of sarcoidosis

(n = 132)

Reports sought for retrieval

(n = 44)

Reports not retrieved

(n = 1)

Reports assessed for eligibility

(n = 43)

Studies included in **review**

(n = 21)

**Identification**

**Screening**

**Included**

Reports excluded: (n = 22 )

No necrotizing lesion

Necrotizing lesion but no CNS sign

PubMed search: *"sarcoidosis* AND (*necrotic* OR *necrotizing* OR *necrosis*) AND (*brain* OR c*erebral* OR *cerebellar OR meninge OR meningeal OR nerve OR headache OR diplopia*)*"*
