# Supplementary material for: Case Report: Necrotizing granulomas in the central nervous system: sarcoidosis masquerading as neurotuberculosis
Source: Front Immunol. 2025 Oct 30;16:1653164. doi: 10.3389/fimmu.2025.1653164 (PMC12611859; doi:10.3389/fimmu.2025.1653164)
Supplement: Supplementary file 3 [file Table2.docx]

**Supplemental file 2 Additional clinical and imaging data, literature review of brain lesions associated with necrotizing sarcoidosis**


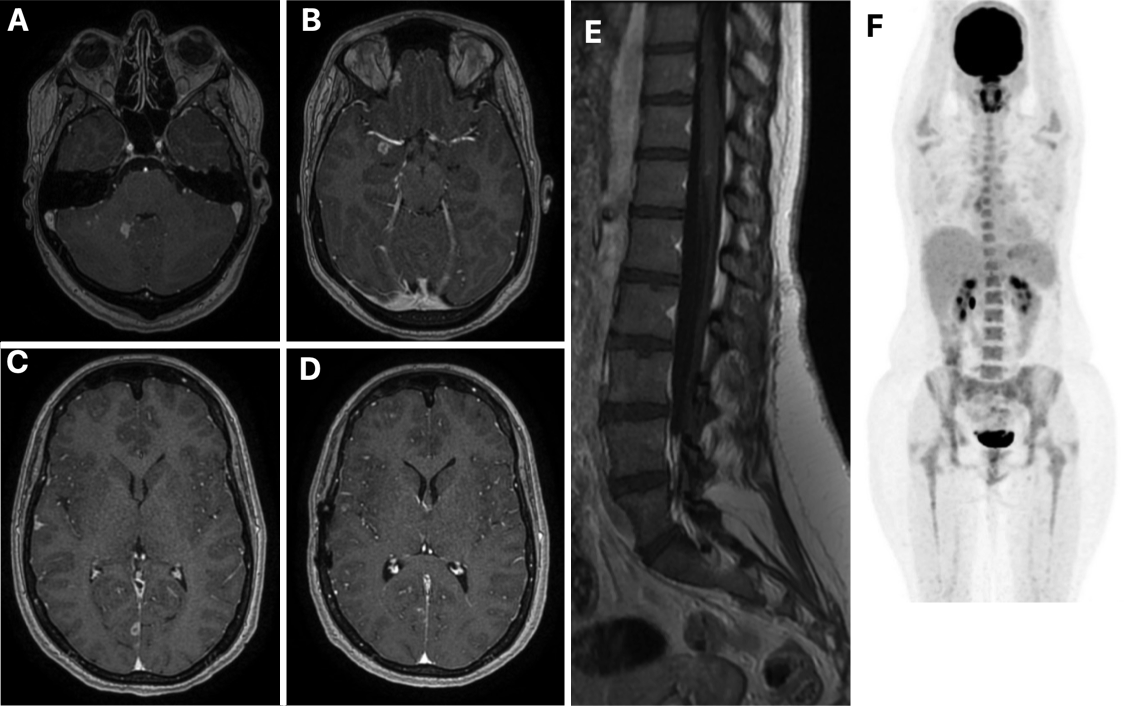


**Figure S1. Additional radiological results (patient 1).** (A) T1-enhanced brain MRI with multiple enhancing lesions in cerebellum, (B) dorsal sylvian vallecula, (C) leptomeninges on temporal and occipital lobes, (D) which vanished after 2 years of treatment. (E) Leptomeningeal enhancing lesion visible at level of terminal conus. (F) FDG-PET shows intense diffuse hypermetabolism of tissues of oral sphere, abnormally pronounced hypermetabolism of spinal cord and diffuse bone marrow hypermetabolism


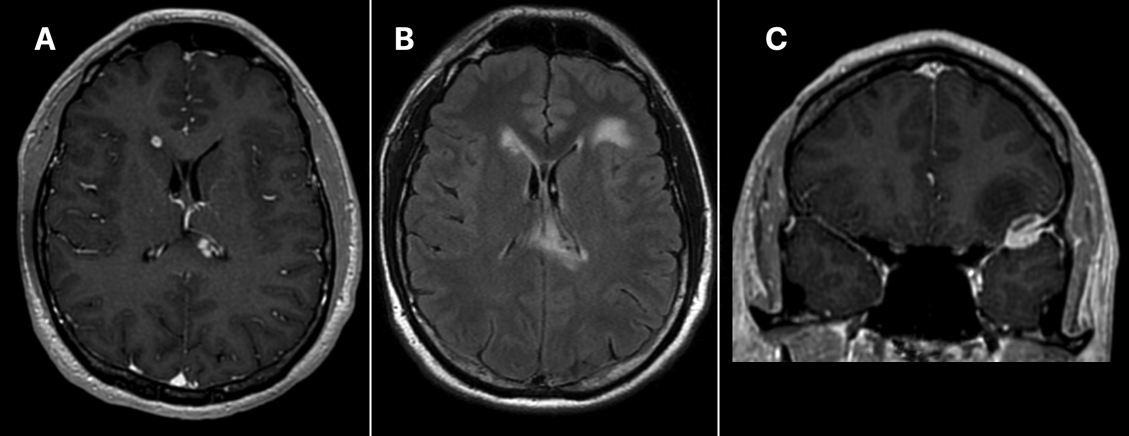


**Figure S2. Additional radiological results (patient 2).** (A) T1-enhanced brain MRI with ring-enhancing periventricular frontal lesion and enhancing left temporal lesion straddling the choroid plexus and ventricle. (B) Perilesional edema is visible on FLAIR sequences acquired in the same plane. (C) Coronal section (T1-enhanced sequence) showing sampled temporal lesion, located in lesser wing of left sphenoid bone

**Table S1 Necrotizing sarcoid granulomatosis (NSG) with CNS involvement.**

| **Ref.** | **Age, sex** | **Known systemic sarcoidosis** | **Symptoms** | **MRI** | **Pathology** | | | **Biopsy location** |
| --- | --- | --- | --- | --- | --- | --- | --- | --- |
|  |  |  |  |  | **Granuloma** | **Necrosis** | **Other** |  |
| [1] | 22, M | Yes (systemic, uveitis, ADP) | Headache, seizures | Multiple nodular lesions | Yes | Necrotizing |  | Parietal |
| [2] | 13, M | Yes | Panuveitis, oculomotor | Pontine mass | Yes | Necrotizing |  | Mass resection |
| [3] | 46, M | Yes | Monocular blindness | No data | Yes | Necrotizing | Fibrosis, inflam. | Left eye |
| [4] | 32, M | No | Seizure | Chiasmatic cistern mass | Yes | Necrotizing |  | Meningeal biopsy |
| [5] | 52, F | Yes (parotid, lung) | 3-month headache, trigeminal | Left temporal mass | Yes | Coagulative |  | Temporal,  partial resection |
| [6] | 51, M | No | 3-month optic, proptosis | Left anterior clinoid process and sphenoid wing | Yes | Mix | Fibrosis, chronic inflam. | Frontotemporal, resection |
| [7]#1 | 36, F | No | Global lower weakness, urinary retention | Medulla hyperintensity | Yes | Necrotizing |  | Left frontal convexity dura and falx |
| [7]#2 | 40, F | No | Hearing loss | Right cerebellopontine angle mass | Yes | Necrotizing | Chronic inflam. | Right retromastoid |
| [7]#3 | 53, M | No | Numbness | Slight expansion of conus medullaris | Yes | Coagulative | Chronic inflam. | Conus medullaris |
| [8] | 35, F | No | Photophobia, diplopia, headache | Tumor in left orbit | Yes | Necrotizing |  | Left orbit |
| [9] | 46, F | No | General weakness, frequent falls | Extensive LM | Yes | Necrotizing |  | No data |
| [10] | 27, M | No | Unconsciousness and convulsion | Multifocal enhancement lesions | Yes | Mix | Fibrosis, chronic inflam. | Left frontotemporal |
| [11] | 32, F | No | Headache | Temporal globular mass | Yes | Necrotizing | Fibrosis | Left subtemporal |
| [12] | 40, M | No | Headache, numbness, diplopia | Petroclival mass | Yes | Necrotizing | Fibro collagenous infiltration | Petroclival mass |
| [13] | 25, M | No | Polyuro-polydipsic syndrome | Supra- and infra-chiasmatic pre- thalamic mass lesion | Yes | Necrotizing | Fibrosis | Cerebral mass |
| [14] | 52, F | No | Headache, aphasia and monocular blindness | Diffuse enhancement of dura | Yes | Necrotizing | Vessel compression by granulomas | Meningeal mass |
| [15] | 12, F | No | Numbness | No data | Yes | Necrotizing | Chronic inflam. | Intradural mass |
| [16] | 44, F | No | Headache, loss of vision | Mass encircling optic nerve | Yes | Necrotizing | Chronic inflam. | Cerebral mass |
| [17] | 31, M |  | Seizure, walking disorders | Multiple nodular periventricular lesions | Yes | Necrotizing | Granulomas in vessels | Pulmonary |
| [18] | 30, M | No | Diplopia, left fourth cranial nerve palsy | No data | Yes | Necrotizing | Fibrosis | Pulmonary |
| [19] | 11, M | No | Spastic paraplegia | No data | Yes | Necrotizing |  | Pulmonary |
| [20] | 31, M | No | Peripheral vertigo, weakness in right lower limb | Meningeal uptake extending to cervical area | Yes | Necrotizing |  | Pulmonary |
| [21] | 30, F | No | Uveitis, left arm deficit | CT scan: frontal nodular lesions | Yes | Necrotizing | Granulomas in vessels | Pulmonary |
| **#1** | 30, F | No | Facial palsy, headache, numbness | Multiple LM lesions | Yes | Necrotizing | Occluded artery | Temporal, meningeal biopsy |
| **#2** | 39, M | No | papillitis bilat., blindness | meninges, CP, ventricle | Yes | Necrotizing |  | Temporal, LM biopsy |

**Abbreviations:** ADP: adenopathy; bilat.: bilateral; CP: choroid plexus; inflam.: inflammation; LM: leptomeningeal.

^1^ however, blood vessels were undersampled.

**References**

[1] Kitajima S, Sakai N, Furuichi K, et al. A case of neurosarcoidosis with necrotizing granuloma expressing angiotensin-converting enzyme. *Mod Rheumatol* 2010; 20: 506–510.

[2] Leiba H, Siatkowski RM, Culbertson WW, et al. Neurosarcoidosis presenting as an intracranial mass in childhood. *J Neuro-Ophthalmol Off J North Am Neuro-Ophthalmol Soc* 1996; 16: 269–273.

[3] Kelley JS, Green WR. Sarcoidosis Involving the Optic Nerve Head. *Arch Ophthalmol* 1973; 89: 486–488.

[4] Grand Rounds--Hammersmith Hospital: Meningeal granulomas: sarcoidosis or tuberculosis? *BMJ* 1995; 310: 517–520.

[5] Strickland-Marmol LB, Fessler RG, Rojiani AM. Necrotizing sarcoid granulomatosis mimicking an intracranial neoplasm: clinicopathologic features and review of the literature. *Mod Pathol Off J U S Can Acad Pathol Inc* 2000; 13: 909–913.

[6] Tobias S, Prayson RA, Lee JH. Necrotizing neurosarcoidosis of the cranial base resembling an en plaque sphenoid wing meningioma: case report. *Neurosurgery* 2002; 51: 1290–1294; discussion 1294.

[7] Markert JM, Powell K, Tubbs RS, et al. Necrotizing neurosarcoid: three cases with varying presentations. *Clin Neuropathol* 2007; 26: 59–67.

[8] Dykhuizen R, Smith C, Kennedy M, et al. Necrotizing sarcoid granulomatosis with extrapulmonary involvement. *Eur Respir J* 1997; 10: 245–247.

[9] Shah U, Carran M. Neurosarcoidosis with Granulomatous Necrosis. *Neurology* 2012; Volume 78: Pages: P03.252.

[10] Wang K, He X, Wang W, et al. Isolated neurosarcoidosis mimicking multifocal meningiomas: a diagnosis pitfall A case report. *Medicine (Baltimore)* 2016; 95: e4994.

[11] Ghozzi A, Azouz H, Chelly I, et al. Unusual presentation of sarcoidosis: solitary intracranial mass lesion mimicking an intracranial neoplasm: a case report. *Pan Afr Med J*; 18. Epub ahead of print 2014. DOI: 10.11604/pamj.2014.18.236.1409.

[12] Valappil A, Sundar S S, Johny M, et al. Intracranial necrotising sarcoid granulomatosis mimicking petroclival meningioma. *BMJ Case Rep* 2022; 15: e247792.

[13] Krenzlin H, Jussen D, Musahl C, et al. A Rare Case of Isolated Cerebral Sarcoidosis Presenting as Suprasellar Mass Lesion with Salt-Wasting Hypopituitarism. *J Neurol Surg Rep* 2015; 76: e140–e145.

[14] Savage NM, Shah H, Alleyne CH, et al. Neurosarcoidosis with necrotising sarcoid granulomatosis mimicking meningiomatosis cerebri: case report and literature search. *Case Rep* 2009; 2009: bcr1120081187–bcr1120081187.

[15] Singh N, Cole S, Krause PJ, et al. Necrotizing sarcoid granulomatosis with extrapulmonary involvement. Clinical, pathologic, ultrastructural, and immunologic features. *Am Rev Respir Dis* 1981; 124: 189–192.

[16] Jennings JW, Rojiani AM, Brem SS, et al. Necrotizing neurosarcoidosis masquerading as a left optic nerve meningioma: case report. *AJNR Am J Neuroradiol* 2002; 23: 660–662.

[17] Ríos Fernández R, Callejas-Rubio JL, Guerrero Fernández M, et al. Sarcoidosis sistémica necrotizante con afectación pulmonar y del sistema nervioso central. *An Med Interna*; 25. Epub ahead of print January 2008. DOI: 10.4321/S0212-71992008000100006.

[18] McArdle DJT, McArdle JP, Jessup P, et al. Necrotizing Sarcoid Granulomatosis: Clinico-Radio-Pathologic Diagnosis. *Am J Med* 2017; 130: e283–e286.

[19] Beach RC, Corrin B, Scopes JW, et al. Necrotizing sarcoid granulomatosis with neurologic lesions in a child. *J Pediatr* 1980; 97: 950–953.

[20] Parejo-Morón AI, Tornero-Divieso ML, Férnandez-Díaz MR, et al. Necrotizing Sarcoid Granulomatosis: A Disease Not to be Forgotten. *Case Rep Med* 2020; 2020: 1–4.

[21] Brevet F, Hachulla É, Courtin Ph, et al. Forme systémique de granulomatose nécrosante sarcoïdosique avec atteinte oculaire, pulmonaire et cérébrale. *Rev Médecine Interne* 1993; 14: 243–248.
